# HIV infection and the implication for COVID‐19 vaccination

**DOI:** 10.1002/puh2.14

**Published:** 2022-07-29

**Authors:** Tope Oyelade, Reynie Purnama Raya, Kamaluddin Latief

**Affiliations:** ^1^ Institute for Liver and Digestive Health Division of Medicine University College London London UK; ^2^ Institute for Global Health Faculty of Population Health Sciences University College London London UK; ^3^ Faculty of Science Universitas ‘Aisyiyah Bandung Bandung Indonesia; ^4^ Global Health and Health Security Department College of Public Health Taipei Medical University Taipei City Taiwan; ^5^ Centre for Family Welfare Faculty of Public Health University of Indonesia Depok Indonesia

**Keywords:** AIDS, COVID‐19, HIV, public health, SARS‐CoV‐2, vaccine

## Abstract

**Abstract:**

Human immunodeficiency virus (HIV) is associated with altered cellular and humoral immune response, especially in patients with an untreated or chronic infection. This may be due to direct and/or indirect HIV viral activities resulting in T‐ and B‐cells dysfunctions. Although still unclear, various studies have proposed that HIV infection may exacerbate the clinical outcomes of COVID‐19. Indeed, COVID‐19 vaccines were developed in record time and have been shown to reduce the severity of COVID‐19 in the general population. These vaccines were also earmarked as a solution to global disruptions caused by the COVID‐19 pandemic. HIV infection has been reported to reduce the efficacy of various other vaccines including those used against *Streptococcus pneumoniae, Clostridium tetani*, and influenza viruses. However, current guidelines for the administration of available COVID‐19 vaccines do not account for the immune‐compromised state of people living with HIV (PLWH). We discuss here the potentials, nature, and implications of this HIV‐induced dampening of the humoral immune response on COVID‐19 vaccines by first reviewing the literature about efficacy of previous vaccines in PLWH, and then assessing the proportion of PLWH included in phase III clinical trials of the COVID‐19 vaccines currently available. The clinical and public health implications as well as suggestions for governments and non‐governmental organizations are also proposed in the context of whether findings on the safety and efficacy of the vaccines could be extended to PLWH.

**Impacts:**

- The human immunodeficiency virus (HIV) is characterized by attenuated humoral immunity that may reduce the efficacy of vaccines in people living with HIV (PLWH). Vaccination against the SARS‐CoV‐2 infection remains the main public health answer to the COVID‐19 pandemic.
- Although no significant safety concerns have been raised regarding the COVID‐19 vaccines in PLWH, the efficacy of these vaccines in PLWH has not received due attention. Indeed, phase III clinical trials for the safety and efficacy of COVID‐19 vaccines involved a significantly low number of PLWH.
- There are major gaps in knowledge on the efficacy of COVID‐19 vaccines in PLWH and until further research is carried out, PLWH should be prioritized along with other at‐risk groups for repeated vaccination and safeguard.

## Graphical abstract

Direct and indirect effect of HIV viraemia on B‐cell population and activity is linked with sub‐optimal efficacy of vaccines in people living with HIV (PLWH) through quantitative or qualitative reduction in elicited immune response (neutralizing antibodies). The proportion of PLWH included in phase III clinical trials of COVID‐19 vaccines is significantly low and extension of findings to this population may be misleading. We discuss here the implication of these factors on COVID‐19 vaccines efficacy in PLWH.

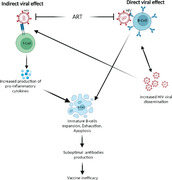

## INTRODUCTION

Human immunodeficiency virus (HIV) is a ribonucleic acid virus that belongs to the lentivirus genus of Retroviridae and etiologically drives the acquired immunodeficiency syndrome (AIDS) which is characterized by a weakened immune response and opportunistic infections [1]. There are two known strains of HIV: type 1 (HIV‐1) and type 2 (HIV‐2). Although both HIV‐1 and HIV‐2 are similar in terms of pathogenic characteristics and tend to progress to AIDS in infected individuals, HIV‐2 is comparatively less virulent and epidemiologically confined to western Africa compared with HIV‐1 which is more globally spread with a higher propensity for progression [2]. Globally, 38 million people were living with HIV between 2020 and June 2021 according to the Joint United Nations Programme on HIV and AIDS (UNAIDS) [3]. Respectively, around 1.5 million and 0.7 million people were newly infected and died from AIDS‐related illnesses during the same period with around 80 million infections so far since the start of the HIV epidemic in 1981 [4]. HIV/AIDS is associated with changes in the physiological function of both the innate and adaptive immune systems, a factor that may predispose people living with HIV (PLWH) to opportunistic infections, especially in patients with high HIV viremia [5].

The coronavirus disease 2019 (COVID‐19) is caused by the severe acute respiratory syndrome coronavirus 2 (SARS‐CoV‐2) which was first discovered in Hubei Province of China in the winter of 2019 [6, 7]. Since its inception, the SARS‐CoV‐2 has infected 425,833,769 people and caused a total of 5,910,094 deaths globally [8], making the SARS‐CoV‐2 so far, the most virulent of the three coronavirus outbreaks in the past decades [9]. This is attributed to the presence of evolutionary advantage from a furin‐like cleavage site on the SARS‐CoV‐2 spike (S) protein which increases the viral‐host cells binding affinity[10]. Indeed, studies have shown that the severity of COVID‐19 and related death is linked with the presence of concomitant morbidities such as chronic obstructive pulmonary disease (COPD), cardiovascular diseases, obesity among others [11, 12, 13]. The COVID‐19 outbreak has been a global challenge, testing the limits of political policies and clinical practices as hospitals around the world struggle to contain the number of infected patients needing hospitalization [14]. This led to the introduction of several measures by governments around the world to curtail the spread of SARS‐CoV‐2 [15, 16]. However, the rapid development of effective vaccines against the virus represents a pivotal stage during the COVID‐19 pandemic and was heralded as a feasible way out of the debilitating social restrictions put in place and their physical and mental consequences [17, 18, 19].

The relationship between HIV and COVID‐19 is still unclear and remains a topical discussion with varied and conflicting reports and hypotheses. While various studies reported an increased risk of severity and death from COVID‐19 in PLWH compared with HIV‐negative individuals [20, 21, 22], other reports contradict these findings [23, 24]. This is mainly due to the heterogeneity in available data as well as the existence of various fundamental confounders that may drive the risk of mortality in PLWH including socio‐economic status, race, antiretroviral therapy (ART) accessibility, and age [25]. Most importantly, the use of ART in PLWH is associated with the restoration of effective immunity and has been linked with a reduced risk of opportunistic infections. Indeed, in a systematic review by Mellor et al., ART‐administered and higher CD4+ T‐cell count in PLWH was associated with a reduced risk of COVID‐19 [26]. However, ART may also lead to severe acute inflammatory response syndrome or residual inflammation which may affect the immune response to vaccination in PLWH [27]. Further, the availability and uptake of ART treatment in certain regions of the world including South‐East Asia and Africa is suboptimal and further confounds an effective global approach to COVID‐19 vaccination [28, 29]. Further, the shock to global health systems due to the COVID‐19 pandemic resulted in interruptions to routine HIV care in the form of reduced ART uptake, loss to follow up as well as social and mental health issues especially in Africa [30, 31]. Put together, these factors present a perfect storm for poorer clinical outcomes of COVID‐19 in PLWH, especially in developing countries with the highest prevalence of HIV cases. Indeed, in recent systematic reviews and meta‐analyses, the risk of severe COVID‐19 was reported to be higher in Africa while the risk of death due to COVID‐19 was higher in North America [20, 32]. Thus, the relationship between COVID‐19 and HIV infection is complex and various unique regional and sub‐populational factors need to be considered on a case‐by‐case and region‐by‐region bases.

Albeit the immune system is dysregulated in PLWH, the implication of this on the COVID‐19 vaccine did not receive the deserved attention and current guidelines including that of the world health organization (WHO) [33] and others recommend that PLWH should be vaccinated similarly to the general population [34, 35]. In this review, we discuss the effect of HIV viremia on vaccine‐dependence immune response with a focus on previous vaccines such as those used against *Streptococcus pneumoniae, Clostridium tetani*, and influenza viral infections. We synthesize the proportion of PLWH included in COVID‐19 vaccine phase 3 clinical trials to assess representation and discuss the implications of this proportion on the possibility of extending the findings of the efficacy of these vaccines to PLWH. The clinical and public health implications of current practices as well as suggestions for governments and non‐governmental organizations are also proposed while future research focuses are highlighted.

## HIV INFECTION AND HUMORAL IMMUNE RESPONSE (B CELLS)

In most people who do not receive effective ART, HIV infection causes persistent viral replication, resulting in variable levels of detectable plasma viraemia. Thus, chronic untreated HIV infection is associated with immune dysfunction usually expressed as loss or exhaustion of T cells including the CD4^+^ and CD8^+^ subtypes. While the loss of T cells in PLWH with uncontrolled viral replication is a hallmark of AIDS and principally drives exacerbation and death [36, 37, 38, 39], HIV infection is also associated with B‐cell dysfunction which may affect the generation of neutralizing antibodies against disease‐causing pathogens [40].

The effect of ongoing HIV replication on B‐cells is thought to be a combination of direct interactions with the virus and indirect interactions associated with a wide range of systemic immune perturbations. Indeed, the first direct interaction between HIV and B‐cells was reported by Schnittman et al. in 1986 [41], opening the door to further investigations of this pathogenic mechanism. Although there is no proof that HIV can replicate in the cytoplasm of B‐cells, there is strong evidence that HIV binds to B‐cells in vivo via interaction between the complement receptor, CD21, and the HIV‐bound antibodies or complements in circulation [42, 43]. Further, other direct binding activities have been reported in both in vivo and in vitro studies involving other B‐cell‐specific HIV‐binding receptors such as the VH3‐family immunoglobulin, C‐type lectin receptors, and DC‐specific ICAM3‐grabbing non‐integrin (DC‐SIGN) [44, 45, 46]. These direct receptor‐dependent interactions are associated with increased dissemination of viral population as well as B‐cell apoptosis and depletion in PLWH. However, the direct interaction between B‐cell and HIV provides an incomplete mechanistic insight into the pathways involved in B‐cells dysfunction driven by HIV viremia.

The indirect HIV effect on B‐cell function is complex and may be expressed in terms of B‐cell hyperactivity, lymphopenia, and exhaustion [40, 47]. The mechanism of HIV‐induced B‐cell hyperactivity has been hypothesized to be linked with dysregulated inflammatory response characterized by increased systemic production of various pro‐inflammatory cytokines such as interleukin‐6 (IL‐6) [48], interleukin‐10 (IL‐10) [49], interferon‐α (IFN‐ α) [50], tumor necrosis factor (TNF) [51] as well as growth factors including the B‐cell activation factor (BAFF) [45] amongst others, all of which are significantly increased in serums of patients with HIV viremia. Further, B‐cell lymphopenia specifically characterized by immature transitional B‐cell expansion is another indirect effect of HIV viremia. Mechanistically, the expansion of immature transitional B‐cells occurs via the decreased systemic level of CD4^+^ T‐cells and associated increased systemic production of IL‐7, a non‐hematopoietic T‐cell homeostatic cytokine capable of inducing the propagation of B‐cell precursor in PLWH [52, 53, 54, 55]. Finally, HIV‐induced exhaustion of B‐cells is characterized by loss of immune function in the form of reduced immunoglobulin generation and is mostly linked with chronic, uncontrolled viral infections. These exhausted B‐cells were first described by Moir et al. who termed them “tissue‐like memory B‐cells” and are associated with increased expression of inhibitory receptors such as the Fc‐receptor‐like 4 (FCRL4), CD20, CD22, CD72, CD85j, CD85k, leukocyte‐associated immunoglobulin‐like receptor 1 and corresponding low expression of B‐cell functional biomarkers including CD21, associated with assimilation of complement‐bound immune complex and CD27, the classic biomarker expressed by active B‐cells capable of somatic hypermutation and immunoglobulin production [56]. The expression of these inhibitory receptors and downregulation of functional biomarkers on B‐cells are characteristic of chronic HIV viraemia and shows a subpopulation of B‐cells exhausted and unable to elicit effective immune response especially in response to pathogens or vaccines. Indeed, some of these effect of HIV viraemia on B‐cells functions are either reversible or unaffected by ART, dependent on stage of HIV infection and whether or not ART was initiated early [40].

**FIGURE 1:**
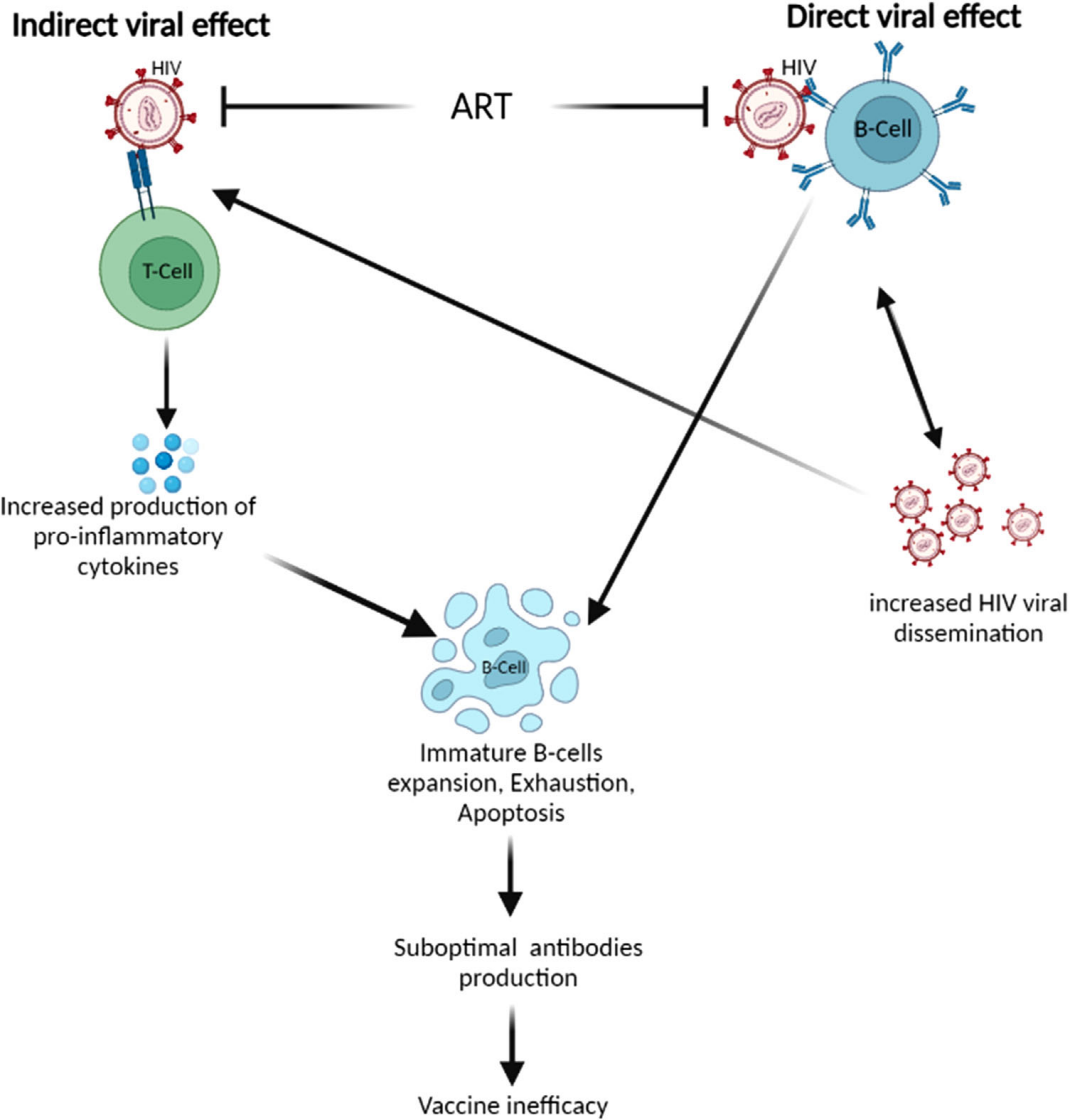
Schematic diagram summarizing the pathways involved in the direct and indirect effect of HIV (human immuno‐deficiency virus) on B‐cells, antibody production, and vaccine efficacy. Abbreviation: ART, anti‐retroviral therapy

In sum, direct receptor binding of HIV to B‐cells has been reported and may result in systemic viral dissemination, B‐cells apoptosis, and depletion while indirect HIV effect is driven via dysregulation of the systemic inflammatory response which may result in B‐cell hyperactivity, exhaustion, expansion of immature B‐cells and lymphopenia (Figure 1). The result of these interactions is a reduced immune response which may affect vaccine efficacy.

## HIV INFECTION AND COVID‐19 VACCINES

The drive to develop vaccines against human‐infecting coronavirus was first proposed during the previous outbreaks of the SARS‐CoV and MERS‐CoV. However, these vaccines were not tested beyond phase I clinical trials [57, 58, 59]. Albeit the development of an effective vaccine against the SARS and MERS coronaviruses (CoV) did not materialize due to various reasons, knowledge from preclinical research and clinical trial outcomes laid the background for the development of the COVID‐19 vaccine [60]. For instance, the importance of the spike protein as a medium of host cell receptor binding [61], membrane fusion, and cytoplasmic invasion/colonization [62]. Further, a study by Pallesen et al. (2017), showed that the receptor‐binding (RBD) and non‐binding domains of the MERS‐CoV spike protein could provide a viable vaccine target against the virus [63]. The molecular bases of this were on the knowledge that compared to other parts of the virus, the S1 (membrane distal) and S2 (membrane proximal) subunits of the spike glycoprotein are a relatively highly conserved part of the CoV makeup [64]. The viability of this work was supported by another work showing that of the four structural proteins expressed, the spike glycoprotein elicited the production of neutralizing antibodies (NAbs) able to attack the CoV viruses in vivo [65, 66, 67, 68]. This wealth of knowledge gathered through works done on the other strains of the coronaviruses drove the search for effective vaccines against the COVID‐19 pandemic as it ravaged populations globally in 2020.

As of January 2022, the number of vaccines against the various strains of the SARS‐CoV‐2 virus still being researched is 334 of which 140 are already in the clinical stage. According to WHO summary information on COVID‐19 vaccine development, 47 (34%) and 23 (17%) of these vaccines were based on protein and RNA subunits as immunogenic platforms, respectively [69]. Importantly, although PLWH were included in phase III clinical trials of the main COVID‐19 vaccines that is, Johnson and Johnson (Ad26.COV2.S), Pfizer BioNTech (BNT162b2), and Moderna, the proportion of this group of patients was significantly low with the lowest proportion reported in the Pfizer trials (0.5%) [70] and the highest observed in the Johnson and Johnson COVID‐19 vaccine (Ad26.COV2.S) trial (2.5%). Also, the product monograph that was supplied with the Ad26.COV2.S warned that it may not elicit a protective immune response in patients with substantial immune suppression or compromise [71].

Table 1 shows the details of phase III clinical trials with completed and published data regarding the assessment of the safety and efficacy of various COVID‐19 vaccines. Importantly, the table contains 10 of the COVID‐19 vaccine brands with publicly available phase III clinical trials data following a guided search of databases. Overall, these vaccines were trialed in 635,826 volunteers including 1412 PLWH, equivalent to 0.2% of the study population. Despite the low representation of PLWH in the study population, the efficacy of the vaccines in terms of reduction in relative risk (RRR) of up to 94% [72] is extrapolated to PLWH who are currently treated the same way as the HIV‐negative population. This is a major limitation considering that PLWH may be at increased risk of severe COVID‐19 and mortality and their compromised immune system may alter the expected protective immunological response to the vaccines.

**TABLE 1:** Description of the currently openly available data for phase III clinical trial of COVID‐19 vaccines

Name of vaccine	Vaccine code	Vaccine platform	Country of production	Sample size	PLWH *n*(%)	References
Pfizer‐BioNTech	BNT162b2	RNA based vaccine	Germany‐USA	37,706	196 (0.52)	Polack et al. (2020)
Johnson & Johnson	Ad26.COV2.S	Viral vector (non‐replicating)	Netherlands ‐ Belgium	39,321	983 (2.5)	Inc, (2021)
Moderna	mRNA‐1273	RNA based vaccine	USA	30,351	179 (0.59)	Baden et al. (2020)
Oxford‐AstraZeneca	ChAdOx1 (AZD1222)	Viral vector (non‐replicating)	United Kingdom	11,636	NR	Voysey et al. (2021)
Sinopharm‐Beijing	BBIBP	Inactivated Virus	China	40,382	NR	Al Kaabi et al. (2021)
Gamaleya (Sputnik V)	Gam‐COVID‐Vac (Sputnik V)	Viral vector (non‐replicating)	Russia	19,866	NR	Logunov et al. (2021)
Sinovac	CoronaVac	Inactivated Virus	Turkey	10,214	NR	Tanriover et al. (2021)
Bharat Biotech (Covaxin)	Covaxin	Inactivated Virus	India	25,798	NR	Ella et al. (2021)
Soberana 02	Soberana 02	Conjugate	Cuba	440,311	NR	Toledo‐Romani et al. (2021)

Abbreviations: HIV, human immunodeficiency virus; NR, not reported; USA, United States of America.

References

Al Kaabi N., Zhang Y., Xia S., et al. Effect of 2 inactivated SARS‐CoV‐2 vaccines on symptomatic COVID‐19 infection in adults: a randomized clinical trial. *JAMA*. 2021;326(1):35–45. https://doi.org/10.1001/jama.2021.8565

Baden LR, El Sahly HM, Essink B, et al. Efficacy and safety of the mRNA‐1273 SARS‐CoV‐2 vaccine. *N Engl J Med*. 2020;384(5):403–416. https://doi.org/10.1056/NEJMoa2035389

Ella R, Reddy S, Blackwelder W, et al. Efficacy, safety, and lot to lot immunogenicity of an inactivated SARS‐CoV‐2 vaccine (BBV152): a, double‐blind, randomised, controlled phase 3 trial. *medRxiv*. 2021. https://doi.org/10.1101/2021.06.30.21259439

Inc, J. *Product Monograph Including Patient Medication Information*. 2021. Retrieved from https://www.janssen.com/canada/:https://covid‐vaccine.canada.ca/info/pdf/janssen‐covid‐19‐vaccine‐pm‐en.pdf

Logunov DY, Dolzhikova IV, Shcheblyakov DV, et al. Safety and efficacy of an rAd26 and rAd5 vector‐based heterologous prime‐boost COVID‐19 vaccine: an interim analysis of a randomised controlled phase 3 trial in Russia. *Lancet*. 2021;397(10275):671–681. https://doi.org/10.1016/S0140‐6736(21)00234‐8

Polack FP, Thomas SJ, Kitchin N, et al. Safety and Efficacy of the BNT162b2 mRNA Covid‐19 Vaccine. *N Engl J Med*. 2020;383(27):2603–2615. https://doi.org/10.1056/NEJMoa2034577

Tanriover MD, Doğanay HL, Akova M, et al. Efficacy and safety of an inactivated whole‐virion SARS‐CoV‐2 vaccine (CoronaVac): interim results of a double‐blind, randomised, placebo‐controlled, phase 3 trial in Turkey. *Lancet*. 2021;398(10296):213–222.

Toledo‐Romani ME, Garcia‐Carmenate M, Silva CV, et al. Efficacy and safety of SOBERANA 02, a COVID‐19 conjugate vaccine in heterologous three‐dose combination. *medRxiv*, 2021. https://doi.org/10.1101/2021.10.31.21265703

Voysey M, Clemens SAC, Madhi SA, et al. Safety and efficacy of the ChAdOx1 nCoV‐19 vaccine (AZD1222) against SARS‐CoV‐2: an interim analysis of four randomised controlled trials in Brazil, South Africa, and the UK. *Lancet*. 2021;397(10269):99–111. https://doi.org/10.1016/S0140‐6736(20)32661‐1

In a study by Zou et al., comparing the immune response to a double dose of inactivated COVID‐19 vaccine (Sinopharm, WIBP‐CorV, Wuhan Institute of Biological Products Co. Ltd.) between 48 PLWH and HIV‐negative controls, the adverse events were similar between the groups after a 70‐day follow‐up period. However, the antibody response dynamic was different between the groups whereby the humoral immune response was measured by days for neutralizing antibodies to attain peak level, binding antibody units per mL (BAU/mL), geometric mean concentration (GMC), geometric mean ELISA units (GMEU) and seroconversion rates, all showed relatively lower immunogenicity of the vaccine in PLWH compared with HIV‐negative controls [73]. On the contrary, in a sub‐study of phase III clinical trial investigating the safety and immunogenicity of the Oxford‐AstraZeneca (ChAdOx1 nCoV‐19/ AZD1222) vaccine in 54 PLWH who were double‐dosed, no difference was found in immunogenicity and safety compared with non‐HIV volunteers. However, this population was all male and all were on ART with well‐controlled HIV viral loads (<50 copies per mL) [74]. Put together, for all studies conducted to assess the safety and efficacy of all the currently available COVID‐19 vaccines with publicly available datasets, the proportion of PLWH included does not provide a significant or confident affirmation that HIV individuals are as responsive to the vaccines as the general population even though they are currently treated as such.

## EFFICACY OF OTHER VACCINES IN PLWH

The effect of HIV infection on vaccine‐induced immune response depends on the physiological integrity of the patient's immune system as determined by the clinical stage of infection and management of viral load through the accessibility of ART. As discussed above, HIV infection, directly and indirectly, affect both the cellular and humoral immune response and may influence the efficacy of vaccines. Because PLWH are exposed to various secondary infections as with the general population, they are consequently recommended for most of the vaccines currently used to prevent these infections. However, previous studies on the efficacy of other vaccines in PLWH have shown that compromised immune function from chronic or untreated HIV infection may result in suboptimal production of neutralizing antibody and by extension reduce protection. Details is presented below.

### Pneumonia vaccines

Invasive pneumococcal disease (IPD), caused by *Streptococcus pneumoniae* and defined as bacterial invasion of blood and cerebrospinal fluid is a major opportunistic infection in PLWH and while ART may help, the risk of infection is still up to 40‐fold greater in PLWH compared with the general population [75, 76]. Thus, pneumococcal vaccination is highly indicated in PLWH and is safe and especially more efficacious in individuals on ART compared with those who are not. Indeed, administration of the pneumococcal vaccines resulted in over 70% risk reduction of reinfection and recurrence of IPD in the general population [77]. However, a phase III clinical trial by Shabir et al. of around 40,000 children showed that while pneumococcal conjugate vaccine administration resulted in a similar quantitative immune response (measured by geometric mean antibody concentrations, GMC), children with HIV showed a relatively lower qualitative humoral immune response (measured by opsonophagocytic assay, OPA) compared with HIV negative children [78]. Effectively, this shows that while antibodies were indeed produced at a detectable level, PLWH may be less functionally vaccinated against the pneumococcal bacteria compared with HIV‐negative individuals. Furthermore, the administration of the 23‐valent pneumococcal polysaccharide vaccine (PPV23), a T‐cell‐independent vaccine platform especially recommended for PLWH has been consistently reported to be suboptimal in preventing IPD in PLWH [79, 80, 81, 82, 83].

### Tetanus vaccine

Lethal infection caused by toxic metabolites of *Clostridium tetani* is preventable by administration of the tetanus toxoid (TT) vaccine which elicits neutralizing antibodies (NAbs) that may persist for up to 70 years in vaccinated individuals [84, 85]. However, a recent report by Dauby et al. investigating TT vaccine persistence in 103 PLWH from within and outside Europe showed the average half‐life of TT‐specific antibodies to be 9.9 years 95% CI: 5.5–50) with more than 50% reduction in half‐life for subjects from outside Europe [86]. This and other studies with similar findings [87, 88, 89, 90] strongly indicate the need for repeated booster TT vaccine administration to maintain protection against *Clostridium tetani* infection in PLWH.

### Influenza vaccine

PLWH are especially susceptible to various respiratory infections including seasonal influenza and the 2009 H1N1 pandemic influenza A (H1N1pdm). Although, influenza remains a major cause of morbidity and mortality globally, the risk of infection in PLWH has been reported to be similar to that of HIV‐negative individuals [91]. However, several studies have shown that HIV infection is associated with an increased risk of severe influenza and mortality, especially in PLWH who also smoke tobacco or those not on ART [91, 92, 93, 94]. Thus, PLWH are clinically considered a higher risk population in terms of complications of influenza and are recommended generally for a yearly dose of influenza vaccines [95]. Indeed, the influenza vaccine is safe in PLWH, various studies have reported a reduced immunogenic response due to HIV infection. For instance, Chadwick et al. showed reduced (44%) quantitative vaccine efficacy (measured by the geometric mean titer of influenza A antigens) in children with HIV compared to those without HIV (70%). Although this difference was not statistically significant [96]. Lyall et al. corroborated these findings in a group of 25 vertically infected children with HIV whereby children with HIV did not produce an effective quantity of antibodies against the influenza virus (measured as a geometric mean titer and protective level) [97]. Put together, these studies showed that PLWH responds significantly less to vaccinations for various infectious agents due to dysregulation of the immune system heralded by HIV infection and AIDS.

### Other vaccines

HIV infection and especially low CD4+ T‐cell count are also linked with an increased risk of meningococcal disease as well as hepatitis. Indeed, PLWH have been reported to show up to a 24‐fold additional risk of carrying the meningococcal disease compared with HIV‐negative individuals [98, 99]. While no safety concerns have been raised regarding the administration of the meningococcal conjugate vaccines in PLWH, various studies have reported reduced immunogenicity in PLWH compared with the general population [100, 101, 102, 103]. Also, with up to 23% of PLWH coinfected with either hepatitis B (HBV) or C (HCV), viral hepatitis is one of the major coinfections of HIV resulting in an increased risk of liver‐related morbidity and mortality [104, 105]. Thus, while there are currently no vaccines against hepatitis C, vaccination against hepatitis B is highly indicated in PLWH [106, 107]. However, a vaccine against HBV using a recombinant HBV virus elicited a relatively reduced immune response especially in PLWH with lower CD4+ T‐cells [108, 109].

## IMPLICATIONS

Although ambiguity still exists regarding the effect of HIV pre‐infection on COVID‐19 outcomes, untreated or chronic HIV infection has been shown to reduce the effect of other vaccines [75, 87, 88, 110]. This is expected especially in patients with uncontrolled HIV infection with compromised cellular and humoral immune responses [111]. Despite various challenges to HIV treatment during the COVID‐19 pandemic [112], COVID‐19 vaccine uptake in PLWH has been reported to be generally high [113], with age, race, injection of drugs, and geographical inaccessibility identified as factors linked with poorer uptake, especially in patients not primarily on ART or other HIV care [114]. Indeed, reduced willingness to be vaccinated or COVID‐19 vaccine hesitancy has been reported in PLWH, especially in younger patients, patients who inject drugs, those of black and ethnic minority groups, and gender diverse individuals [114, 115]. The implication of low representation of PLWH in phase 3 clinical trial of the vaccines and anxiety about safety have been proposed as contributory factors [116] and warrants further investigations. For instance, studies should address whether vaccine uptake may be improved in PLWH if more data is provided regarding the efficacy and safety in this sub‐population.

Nevertheless. considering the possibility of attenuated immune response to vaccines in some PLWH, the efficacy of COVID‐19 may need to be validated in this subpopulation, and vaccination guidelines may need to be altered following rigorous clinical consideration of the various factors that may attenuate an effective immune response in PLWH. Steps to be considered may include the modification of the vaccine platform, dosage, or repeated booster vaccination [117] to attain a protective level of neutralizing antibodies able to offer the required protection against SARS‐CoV‐2.

## RECOMMENDATIONS

Although the clinical implication of HIV infection on COVID‐19 severity and mortality is still unclear, future studies should focus on clarifying the effect of co‐infection possibly by controlling for ART, and other confounding factors such as age, sex, and comorbidities that may drive the clinical course of COVID‐19 in PLWH. As a research priority, further clinical trials are needed to clearly define the safety and efficacy of COVID‐19 vaccine in PLWH with low or high HIV viremia, in terms of elicited production of a protective level of neutralizing antibodies. This will help clarify the possible need for and those subpopulations of PLWH that may require modification to the current dosage or repeated vaccination if the half‐life of the vaccines is found to be relatively shorter due to high viraemia as reported in the TT vaccine. Special focus should be directed towards low‐ and middle‐income countries with a disproportionately high prevalence of HIV cases, where lower ART availability and uptake have been reported with corresponding increased risk of COVID‐19 severity [20, 32].

## CONCLUSIONS

HIV infection, directly and indirectly, alters the humoral immune response, especially in patients with uncontrolled viral load. The previous coronavirus pandemic, especially the SARS‐CoV and MERS‐CoV outbreaks provided a platform for rapid development of effective COVID‐19 vaccines which are safe and efficacious in the general population. However, the studies on the assessment of efficacy did not include a sufficient population of PLWH. Thus, while the currently available vaccines may be safe for use by PLWH, the efficacy in this population needs further investigation. Especially because some vaccines have been reported to elicit insufficient immunogenicity and preventive neutralizing antibodies in PLWH compared with the general population. Future studies should focus on understanding the dynamics of humoral immune response to COVID‐19 vaccines in PLWH to guide effective policies and practices.

## CONFLICT OF INTEREST

The authors declare no conflict of interest.

## AUTHOR CONTRIBUTIONS

Tope Oyelade: Conceptualization, data curation, formal analysis, investigation, methodology, project administration, resources, software, supervision, validation, visualization, writing – original draft, and writing – review & editing. Reynie Purnama Raya: Data curation, investigation, validation, writing – original draft, and writing – review & editing. Kamaluddin Latief: Investigation, writing – original draft, and writing – review & editing. All authors have read and agreed to the submitted/published version of the manuscript.

## ETHICS STATEMENT

Ethics statement is not available because this is a review article.

## Data Availability

Data sharing is not applicable to this article as no new data were created or analyzed in this study.
